# Bottom-Up Formation of Dodecane-in-Water Nanoemulsions from Hydrothermal Homogeneous Solutions**

**DOI:** 10.1002/anie.201301403

**Published:** 2013-05-13

**Authors:** Shigeru Deguchi, Nao Ifuku

**Affiliations:** Institute of Biogeosciences, Japan Agency for Marine-Earth Science and Technology (JAMSTEC)2-15 Natsushima-cho, Yokosuka 237-0061 (Japan) and Graduate School of Nanobioscience, Yokohama City University22-1 Seto, Kanazawa-ku, Yokohama 236-0027 (Japan) E-mail: shigeru.deguchi@jamstec.go.jp

**Keywords:** colloids, nanoemulsions, nanoparticles, phase transitions

Oil and water do not mix, but often they are made to mix in the form of emulsions by dispersing either component as fine droplets in the other. Emulsions are widely used in such industries as food, pharmaceutical, cosmetic, chemical, agricultural, print/ink, and petroleum.^1^ In recent years, considerable interest has emerged in emulsions containing droplets in the size range between 20 nm and 200 nm.^2^ Such emulsions, the so-called nanoemulsions, differ from macroemulsions in several aspects. Owing to the small droplet size, nanoemulsions are transparent or translucent, and creaming or sedimentation is significantly slower. The small droplet size also has potential benefits for developing a new realm of applications such as pharmaceutical and cosmetic formulations2b, 3 as well as reactors for synthesizing nanomaterials.2b, 4

Emulsions are usually prepared by top-down processes, in which external forces are applied to water/oil/surfactant mixtures to deform and disrupt large droplets into smaller ones. The deformation of the droplet, however, is opposed by the Laplace pressure that is inversely proportional to the droplet size.^1^ Thus, the disruption of the droplet becomes increasingly difficult as the size becomes smaller and the applied energy is rather dissipated as heat.2a,c, 5 Alternatively, the phase inversion temperature (PIT) method has been developed.^1^–^2^ PIT is the temperature at which the spontaneous curvature of a surfactant film at the oil/water interface is zero and the interfacial tension is extremely low.2b When emulsification is performed at PIT, nanosized droplets are easily formed owing to the near-zero interfacial energy.^6^ However, the PIT method can only be used with polyoxyethylene-type nonionic surfactants.^1^

Nanosized solid particles are usually prepared by bottom-up processes.^7^ Unlike the top-down process, the bottom-up process starts with homogeneous solutions and solutes are allowed to assemble to form nanoparticles. It is plain to see that the bottom-up approach is also preferable for fabricating nanosized liquid droplets, but homogeneous solutions of oil and water need to be prepared.

Water near its gas/liquid critical point (*T*_c_=374 °C, *P*_c_=22.1 MPa) exhibits properties that are remarkably different from those at ambient conditions, presumably because high thermal energy suppresses clustering of water molecules.^8^ For example, the dielectric constant of water, which is approximately 80 at ambient conditions, decreases to 2 at 400 °C and 25 MPa (Figure S1 in the Supporting Information), and this value is comparable to those of typical hydrocarbons.^9^ Accordingly, a wide variety of hydrocarbons become freely miscible with water.^10^ We found this opens up a whole new opportunity for emulsification, where oil droplets are formed in a bottom-up manner by phase separation of hydrothermal homogeneous solutions after a rapid and deep-temperature quench (i.e. from very high temperature to low temperature). The process that we call MAGIQ (*m*onodisperse n*a*nodroplet *g*eneration *i*n *q*uenched hydrothermal solution) was applied for emulsifying dodecane in water.

The concept of MAGIQ is illustrated by in situ microscopic images in Figure 1, which were taken on an optical microscope with a high-temperature and high-pressure chamber.^11^ When coarse droplets of dodecane in water were heated in the chamber under a constant pressure of 25 MPa, they started to shrink above approximately 330 °C (Figure 1 a–c). The system eventually entered into a one-phase regime at around 337 °C and the droplets disappeared completely to give a homogeneous solution (Figure 1 d). The system phase-separated again when the solution was cooled, and dodecane droplets having a fairly uniform size reappeared (Figure 1 e), which increased in size as it was cooled further (Figure 1 f). In MAGIQ, a deep-temperature quench is applied to homogeneous solutions so that they phase-separate rapidly to give nanosized droplets.

**Figure 1:**
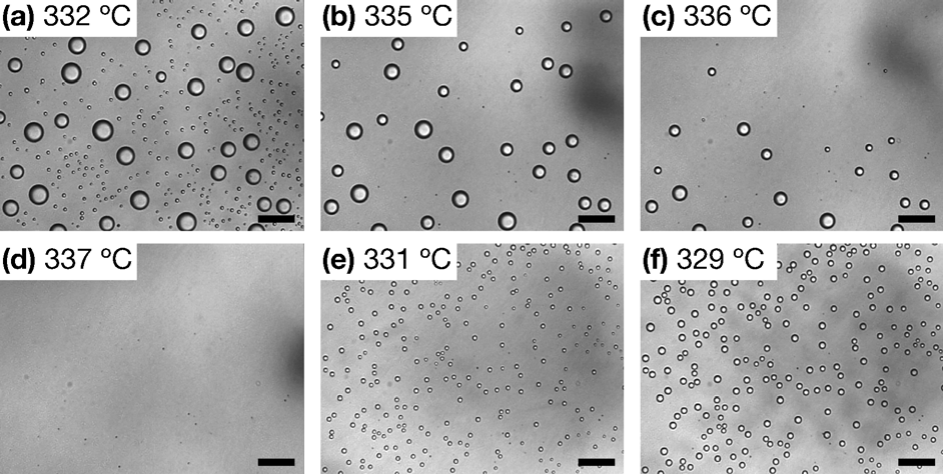
a–d) In situ optical microscopy images of droplets of dodecane in water taken at a constant pressure of 25 MPa. Images were taken while heating the mixture at 11.6 °C min^−1^. e, f) Formation of dodecane droplets upon cooling from (d) at 4.5 °C min^−1^. Scale bars represent 50 μm. Movies showing the processes are available as Supporting Information (Movies S1 and S2).

A flow-type instrument was developed to perform MAGIQ (Figure 2 and Figure S2 in the Supporting Information). Water was heated in a preheat coil under pressure, to which dodecane was added at position T1 (Figure 2). The mixture was kept at high temperature to obtain a homogeneous solution while passing through a mixing tube. The solution was rapidly quenched to induce phase separation by injection of cold water containing a nonionic surfactant, polyoxyethylene(10)oleyl ether (Brij 97), and a cooling coil. The emulsion thus obtained was depressurized and collected at the outlet of a back-pressure regulator that controlled the operating pressure. In this study, the net flow rate of water and dodecane was fixed at 10.0 mL min^−1^, and cold water containing Brij 97 was injected at 10 mL min^−1^. At a total net flow rate of 20 mL min^−1^, 1.2 L of emulsions can be produced in 1 h. The pressure was kept constant at 25 MPa.

**Figure 2:**
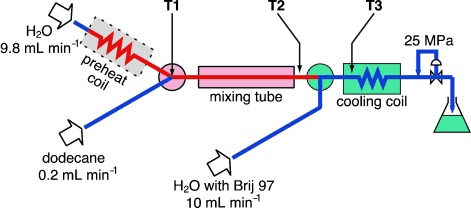
Schematic representation of a flow-type instrument developed in this work. T1, T2, and T3 represent thermocouples. Detailed Scheme of the instrument is available in Figure S2.

We first examined the effect of Brij 97 concentration on emulsification by MAGIQ (Figure 3). The temperatures of the preheat coil and mixing tube were set to 440 °C, which is the highest operating temperature of the instrument. Water and dodecane were fed at a rate of 9.8 and 0.2 mL min^−1^, respectively. The measured fluid temperature at the exit of the mixing tube (T2) was 404 °C, ensuring that mixing of water and hydrocarbons took place in the one-phase regime (Figure 1 and Figure S3 in the Supporting Information). The estimated residence time of the mixture in the mixing tube was 4.5 seconds. The temperature of the solution dropped to 56 °C at T3 within 1.4 seconds, giving a calculated quench rate of 235 °C s^−1^. The variation of the measured fluid temperature at T2 and T3 during experiments was ±1 °C and ±5 °C, respectively. The surfactant concentration was controlled by changing its concentration in the cold water used for quenching. The resulting oil-in-water emulsions contained 1 vol % dodecane.

**Figure 3:**
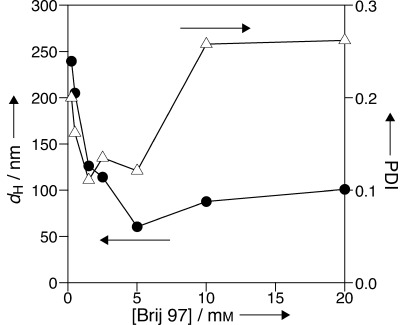
Change of the average hydrodynamic diameter (*d*_H_; •) and the polydispersity index (PDI; ▵) of dodecane droplets as a function of Brij 97 concentration. Error bars, which were calculated as standard deviations of three successive measurements, were smaller than the plot symbols.

Emulsions that were obtained when a homogeneous solution was quenched with water without Brij 97 were unstable and quickly separated. Quenching the solution in the presence of Brij 97 (0.25 mm), however, resulted in a stable oil/water emulsion containing dodecane droplets having an average hydrodynamic diameter (*d*_H_) of 240 nm. The *d*_H_ value decreased with the concentration of Brij 97, and a translucent emulsion containing droplets with a *d*_H_ of 61 nm was obtained with 5 mm Brij 97 (Figure S4 in the Supporting Information). Further increasing the Brij 97 concentration led to an increase of the *d*_H_ value (88 and 101 nm with 10 and 20 mm Brij 97, respectively). Monodisperse droplets, with a polydispersity index (PDI) below 0.2,^12^ were obtained at Brij 97 concentrations below 5 mm, whereas the droplets obtained at higher Brij 97 concentrations had broader size distributions (Figure S4).

The *d*_H_ value of the dodecane droplets obtained with 5 mm Brij 97 increased to 202 nm after storage for five days at room temperature, and remained essentially unchanged thereafter up to one month (Figure S5 in the Supporting Information). Large droplets were formed at the expense of small droplets and the size distribution of the droplets became narrower during initial coarsening, thus suggesting that coarsening was mainly driven by Ostwald ripening and slowed down once the droplet became monodisperse.^1^, ^13^

The temperature at which dodecane and water were mixed was critical for successful nanoemulsification by MAGIQ. Figure 4 a shows the *d*_H_ values of dodecane droplets in oil/water emulsions (1 vol % dodecane) obtained at different mixing temperatures; the mixing temperature is defined as the fluid temperature at the exit of the mixing tube (T2). The concentration of Brij 97 was fixed at 5 mm, which gave the smallest *d*_H_ value in Figure 3. Emulsification was observed even at 30 °C, because dodecane droplets were disrupted at the back-pressure regulator, where the two-phase dodecane/water mixture passed through a narrow slit. The emulsion was very unstable, and the *d*_H_ increased from 486 nm to 1244 nm in 12 min, which lead to a large error bar in Figure 4 a. Similar unstable and coarse emulsions were obtained at mixing temperatures up to 344 °C. A stable emulsion that did not change size during dynamic light-scattering (DLS) measurements was obtained only when the mixing temperature was raised to 373 °C, and a further increase of the mixing temperature resulted in a steep decrease in *d*_H_ and PDI.

**Figure 4:**
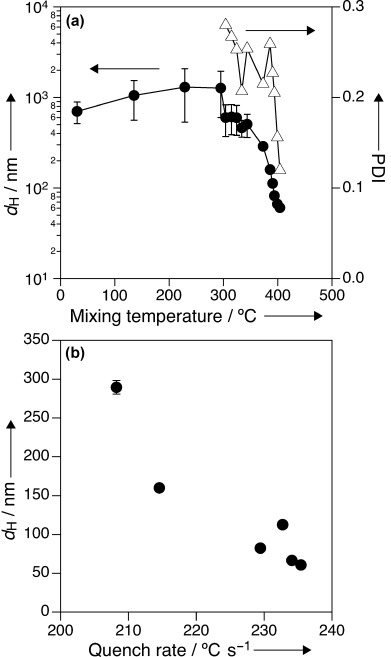
a) Change of *d*_H_ (•) and PDI (▵) of dodecane droplets as a function of the mixing temperature. Error bars represent standard deviations of three successive measurements. b) Relationship between *d*_H_ and the quenching rate for nanoemulsions obtained at *T*_T2_>373 °C.

Clearly, there was a threshold mixing temperature for the formation of stable nanoemulsions, which was 373 °C in the present case. The result highlights the importance of mixing water and dodecane in a one-phase regime in performing MAGIQ, and in turn suggests that a homogeneous solution of water and dodecane was obtained when the mixing temperature was kept above 373 °C. Note that the mixing temperature here represents the fluid temperature at the outlet of the mixing tube and not the exact temperature at which droplet formation (phase separation) occurred.

In addition to the mixing temperature, the quenching rate also affected the final droplet size. We found a good correlation between the quenching rate and the *d*_H_ value obtained above 373 °C (T2), and the *d*_H_ value decreased rapidly with the quench rate (Figure 4 b).

According to the phase diagram (Figure S3), dodecane and water become freely miscible above approximately 363 °C at 25 MPa,10a thus suggesting that MAGIQ should be applicable for emulsification with different dodecane contents. This was verified by applying MAGIQ for emulsifying 0.5, 2, and 10 vol % dodecane in water. Experiments were performed by setting the temperatures of the preheat coil and mixing tube to 440 °C. The oil content in the final mixtures was controlled by the ratio of the flow rates of water and dodecane, while keeping the net flow rate constant at 10 mL min^−1^.

When the concentration of Brij 97 was varied, the *d*_H_ value decreased with increasing Brij 97 concentration and passed minima, just as we observed in the experiments using 1 vol % dodecane (Figure 3). The smallest *d*_H_ value was obtained with 4 mm Brij 97 for 0.5 vol % dodecane (*d*_H_=53 nm) and 12.5 mm Brij 97 for 2 vol % dodecane (*d*_H_=110 nm). In the case of emulsification of 10 vol % dodecane, we were not able to determine the optimal concentration of Brij 97 at which the smallest *d*_H_ value was obtained, because the operating pressure of the instrument became unstable when the concentration of Brij 97 in the water for quenching was increased above 50 mm. The reason for the instability is not clear, but it might be ascribed to the increased viscosity of water containing high concentrations of Brij 97. Nonetheless, a *d*_H_ of 234 nm was obtained with 20 mm Brij 97.

One of the concerns associated with MAGIQ is thermal decomposition of hydrocarbons. It was reported that 24 % of dodecane was thermally cracked when its mixture with water was treated at 425 °C for 30 min.^14^ We observed shorter alkanes and alkenes in dodecane after thermal treatment in the flow-type instrument, but the degree of decomposition was less than 1 % (Figure S6 in the Supporting Information). The result demonstrates a distinct advantage of the flow-type instrument, in which hydrocarbons are exposed to high temperature only briefly during emulsification.

Our results show monodisperse nanodroplets of dodecane were formed in a bottom-up manner when its hydrothermal homogeneous solution was quenched. Considering the depth of the quench and the monodispersity of the droplets, it is likely that the droplets were formed by spinodal decomposition of the solution.^15^ If the droplet formation is governed by the phase-separation dynamics alone, the droplet size should depend on the dodecane content and the rate and depth of the quench, but not on the Brij 97 concentration. Coalescence of the droplets that were initially formed by spinodal decomposition possibly occurred before the Brij 97 molecules adsorbed to the droplet surface and stabilized them. We also observed an increase of the droplet size at higher Brij 97 concentrations accompanied with a broader size distribution. For emulsification of 1 vol % dodecane, the concentration of dodecane in homogeneous solution in water is 44 mm. It could be hypothesized that, in the presence of 10 and 20 mm Brij 97, phase-separation dynamics of ternary mixtures (dodecane/water/Brij97), not pseudo-binary mixtures (dodecane/water with a small amount of Brij 97), would have been responsible for the droplet formation.

In summary, a novel bottom-up emulsification process, which we call MAGIQ, was developed. Quenching hydrothermal homogeneous solutions of dodecane led to the formation of monodisperse nanodroplets. It appears that the droplets are initially formed by spinodal decomposition of the solution, and the final droplet size is determined by the interplay of the phase-separation dynamics, the coalescence kinetics, and the adsorption kinetics of Brij 97. The process was extremely rapid, taking less than 10 seconds from injection to recovery. The flow-type instrument minimized thermal decomposition of dodecane.

Since various linear hydrocarbons (from methane to icosane), a branched hydrocarbon (squalane), an aromatic hydrocarbon (benzene), and various gasses (e.g., O_2_, H_2_, and CO_2_) also become freely miscible with water in a similar temperature and pressure range,^10^, ^16^ MAGIQ should be applicable for emulsifying a wide variety of hydrocarbons or for generating nanobubbles in water. MAGIQ can also be applied for preparing water-in-oil nanoemulsions by injecting water to hot and compressed hydrocarbons, but appropriate measures would have to be implemented to prevent thermal decomposition of the hydrocarbons. It would be interesting to establish composition–temperature phase diagrams of such mixtures, which are crucial in understanding the droplet formation mechanism in MAGIQ, such as the exact temperature at which droplet formation occurs, but which are not available at present. Unlike the PIT method, various types of surfactants can, in principle, be used in MAGIQ, because the major role of the surfactant is droplet stabilization and it has little to do with the droplet formation.

Advantages of supercritical water (SCW) as a medium to synthesize metal oxide nanoparticles have been demonstrated.^17^ When it comes to organic compounds, however, the use of SCW has mostly been limited to their decomposition.^18^ This work is the first demonstration that the unique solvent properties of SCW can also be used for producing organic nanostructures. Besides, SCW does exist in nature, for example in deep-sea hydrothermal vents,^19^ and geochemical implications of very different physical chemistry in SCW have been mooted. These include the formation of hydrothermal ore deposits8a and the fate of plant deposits.^20^ A steep thermocline similar to that used in MAGIQ is formed around the hydrothermal vent, and its relevance for the origin of life was suggested.^21^ The mechanism behind MAGIQ may also have implications for oil-related geochemical processes, such as the formation of oil fields.

## Supplementary material

As a service to our authors and readers, this journal provides supporting information supplied by the authors. Such materials are peer reviewed and may be re-organized for online delivery, but are not copy-edited or typeset. Technical support issues arising from supporting information (other than missing files) should be addressed to the authors.
