# Supplementary material for: Bottom-Up Formation of Dodecane-in-Water Nanoemulsions from Hydrothermal Homogeneous Solutions
Source: Angew Chem Int Ed Engl. 2013 May 13;52(25):6409–12. doi: 10.1002/anie.201301403 (PMC3734699; doi:10.1002/anie.201301403)
Supplement: Supplementary file 1 [file anie0052-6409-SD1.pdf]

Angewandte Chemie
Eine Zeitschrift der Gesellschaft Deutscher Chemiker

Supporting Information

© Wiley-VCH 2013

69451 Weinheim, Germany

Bottom-Up Formation of Dodecane-in-Water Nanoemulsions from Hydrothermal Homogeneous Solutions**

Shigeru Deguchi and Nao Ifuku*

anie_201301403_sm_miscellaneous_information.pdf
anie_201301403_sm_movie_s1.mp4
anie_201301403_sm_movie_s2.mp4

Experimental Section

Supplementary Figures

Figure S1. Change of dielectric constant of water.
Figure S2. Schematic representation of the flow type instrument.
Figure S3. Critical curve for a binary mixture of water and dodecane.
Figure S4. Nanoemulsion obtained by MAGIQ.
Figure S5. Storage stability of nanoemulsion.
Figure S6. Thermal decomposition of dodecane after brief exposure to high temperature.

Supplementary Movies

Movie S1. *In situ* microscopic observation of dodecane droplets in water at high temperature and high pressure. The movie was recorded while heating the mixture at 11.6 ºC/min. Scale bar represents 50 μm. The movie has been edited to 10 times the original playback rate.

Movie S2. *In situ* microscopic observation of hydrothermal dodecane/water solution upon cooling. The movie was recorded while cooling the mixture at 4.5 ºC/min. Scale bar represents 50 μm. The movie has been edited to 5 times the original playback rate.

Experimental Section

Materials. Dodecane (>99% pure) was obtained from Wako Pure Chemical Industries, Ltd. (Osaka, Japan). A technical-grade nonionic surfactant, Brij 97 (polyoxyethylene 10 oleoyl ether) was obtained from Aldrich. Critical micelle concentration of the surfactant was reported to be 0.217 mM.[1] Calculated hydrophilic lipophilic balance (HLB) value of the surfactant is 12.4 according to the manufacturer. Millipore water was used throughout the work. All the chemicals were used without further purification.

In situ high-resolution optical microscopy at high temperature and high pressure. Phase behavior of a dodecane/water mixture was examined by an optical microscope equipped with a high-temperature and high-pressure chamber.[2] The system allows *in situ* observation of a specimen in water at temperatures and pressures above the critical point of water up to 400 ºC and 35 MPa with an optical resolution of 2 µm. A detailed description of the instrument and its operation can be found elsewhere.[2] An emulsion containing 1 vol% dodecane and 5 mM Brij 97 in water was prepared with sonication. The emulsion was introduced into the chamber, pressurized to 25 MPa, heated until the system became homogeneous at around ~340ºC, and cooled to room temperature. The sample was heated again, and dodecane droplets on the surface of the lower optical window were examined.

Dynamic light scattering. Hydrodynamic diameter of droplets in emulsions was measured by dynamic light scattering (DLS) on a Fiber-Optics Particle Analyzer FPAR-1000 (Otsuka Electronics, Osaka, Japan) equipped with a solid-state laser (λ = 658 nm, 30 mW). The measurements were done at 25.0 ± 0.1 ºC.

Thermal decomposition of dodecane. Dodecane was mixed with water while setting the temperatures of the preheat coil and the mixing tube to 440 ºC, and the solution was quenched by mixing with water without Brij 97. The dodecane phase was separated from the aqueous phase, dried with anhydrous Na_2SO_4, and subjected to gas chromatography–mass spectroscopy (GC-MS) analysis on an HP5973 Gas Chromatograph (Agilent Technologies, Santa Clara, USA) connected to an HP5973 Mass Spectrometer (Agilent Technologies) using a (5% phenyl)-methylpolysiloxane stationary phase.

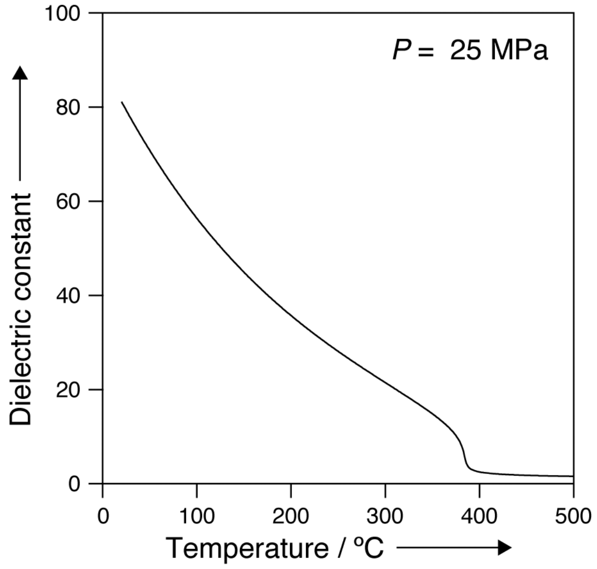

Figure S1. Change of dielectric constant of water as a function of temperature under a constant pressure of 25 MPa.[3]

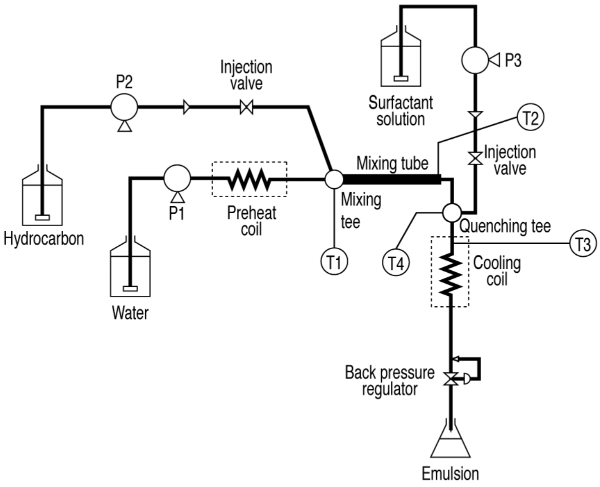

Figure S2. Schematic representation of the flow type instrument developed in this work. It consisted of three pumps (P1, P2, and P3), a preheat coil, a mixing tee, a mixing tube, a quenching tee, a cooling coil, and a back-pressure regulator. The preheat coil and mixing tube, which were made of tubes (ID 1.74 mm) made of Hastelloy C22, were heated by electronic resistive heaters. The mixing tube was a 31.4 cm long straight tube and had an inner volume of 1 cm^3. Cooling coil was immersed in a water bath that was set to 13 ºC. Temperatures were monitored by four thermocouples (T1, T2, T3, and T4). T1 measured the temperature of the mixing tee, whereas T2, T3, T4 directly measured the temperature of the fluid in the instrument. Operating pressure, which was kept constant at 25 MPa in this work, was controlled by the back-pressure regulator located at the end of the flow line. The instrument was manufactured by AKICO Co., Ltd. (Tokyo, Japan).

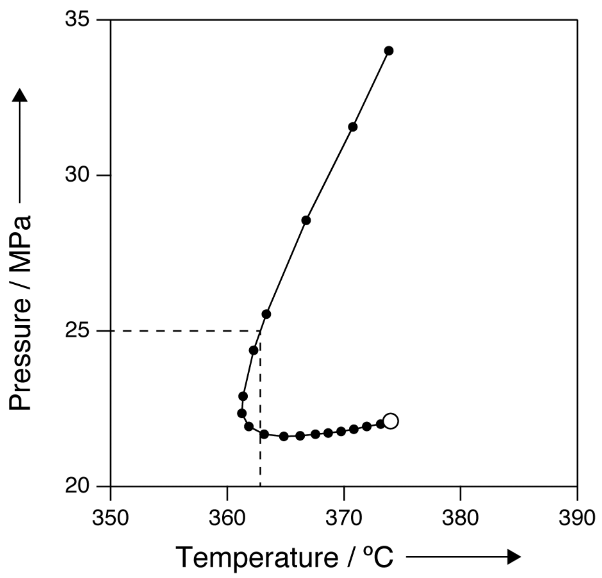

Figure S3. Critical curve for a binary mixture of water and dodecane (taken from ref. [4]). One phase region appears on the right hand side of the curve. Open circle represents the gas/liquid critical point of water (T_c = 374 ºC, P_c = 22.1 MPa). Water and dodecane become freely miscible above ~363 ºC at 25 MPa (indicated by a dotted line).

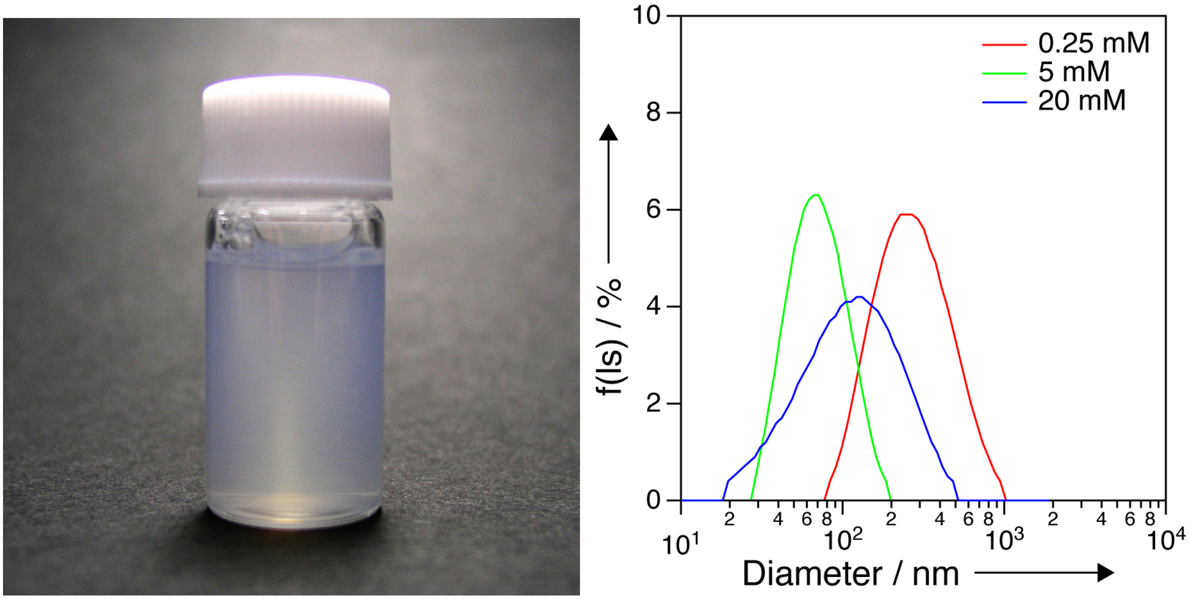

Figure S4. Nanoemulsion obtained by MAGIQ. A photograph of the nanoemulsion obtained with 5 mM Brij 97 (left), and size distribution of dodecane droplets obtained with various concentrations of Brij 97, which was measured by DLS (right). The distribution was obtained by analyzing a measured autocorrelation function by CONTIN.[5]

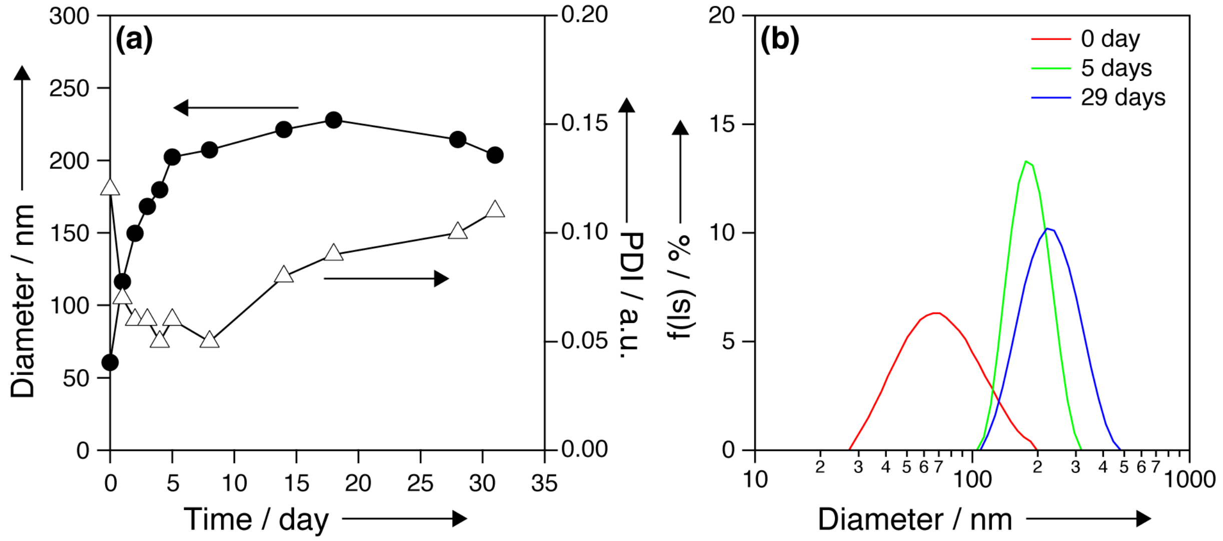

Figure S5. Stability of nanoemulsions obtained by MAGIQ after storage at room temperature. Change of hydrodynamic diameter (closed circles) and polydispersity index (open triangles) (a) and size distribution (b) of dodecane droplets measured by DLS. An average hydrodynamic diameter was obtained by analyzing a measured autocorrelation function by the cumulant method,[6] while CONTIN[5] was used to obtain size distribution. Droplets of two different sizes, one at small size range and one at large size range, should emerge during Ostwald ripening. It is likely that weak scattered light from smaller droplets was masked out by intense scattered light from larger droplets in the DLS measurements.

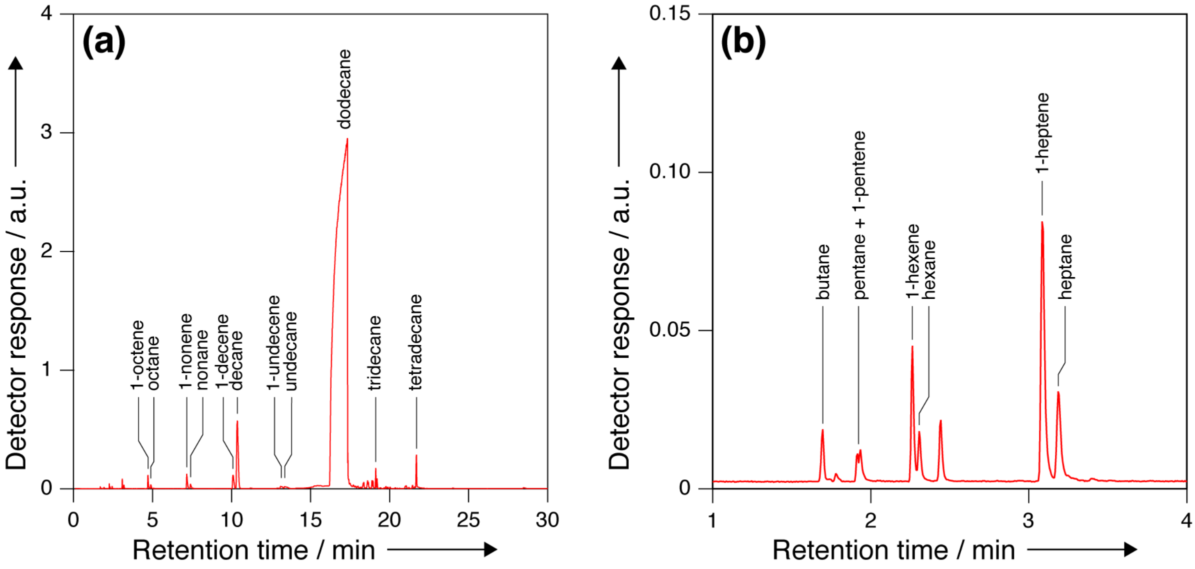

Figure S6. GC traces of dodecane after hydrothermal treatment at 440ºC. Tridecane and tetradecane are impurities, and were found in dodecane before the treatment.

References

[1] C. Klammt, D. Schwarz, K. Fendler, W. Haase, V. Dötsch, F. Bernhard, *FEBS J.* **2005**, *272*, 6024.

[2] a) S. Deguchi, K. Tsujii, *Rev. Sci. Instrum.* **2002**, *73*, 3938; b) S. Mukai, S. Deguchi, K. Tsujii, *Colloids Surf., A* **2006**, *282-283*, 483.

[3] NIST Scientific and Technical Databases: NIST/ASME STEAM Properties Database, Version 2.21, **2004**.

[4] E. Brunner, *J. Chem. Thermodynamics* **1990**, *22*, 335.

[5] S. W. Provencher, *Makromol. Chem.* **1979**, *180*, 201.

[6] D. E. Koppel, *J. Chem. Phys.* **1972**, *57*, 4814.
